# A Novel Technique of Proximal Humerus Fixation

**DOI:** 10.7759/cureus.7706

**Published:** 2020-04-17

**Authors:** MN Baig, Megan Diack, Ben Murphy, Kenneth Kaar

**Affiliations:** 1 Orthopaedics, University Hospital Galway, Galway, IRL; 2 Trauma & Orthopaedics, University Hospital Galway, Galway, IRL

**Keywords:** proximal humerus, percutaneous, anchor fixation

## Abstract

Proximal humerus fractures account for 4%-6% of all the fracture presentations. There are many ways of treating different kinds of proximal humerus fractures. We describe the percutaneous fixation of proximal humerus which is not widely used but is a cost effective, less invasive and comparable to other techniques with regard to results.

## Introduction

The proximal humerus fractures can present in young population with high energy trauma and in elderly population with low energy trauma. The proximal humerus fractures can be treated conservatively as well as operatively depending on multiple variables [1]. In the operative fixation methods most popular are the open reduction internal fixation. We describe our practice of percutaneous fixation which is slightly different than generally used percutaneous fixation.

## Technical report

**Figure 1:**
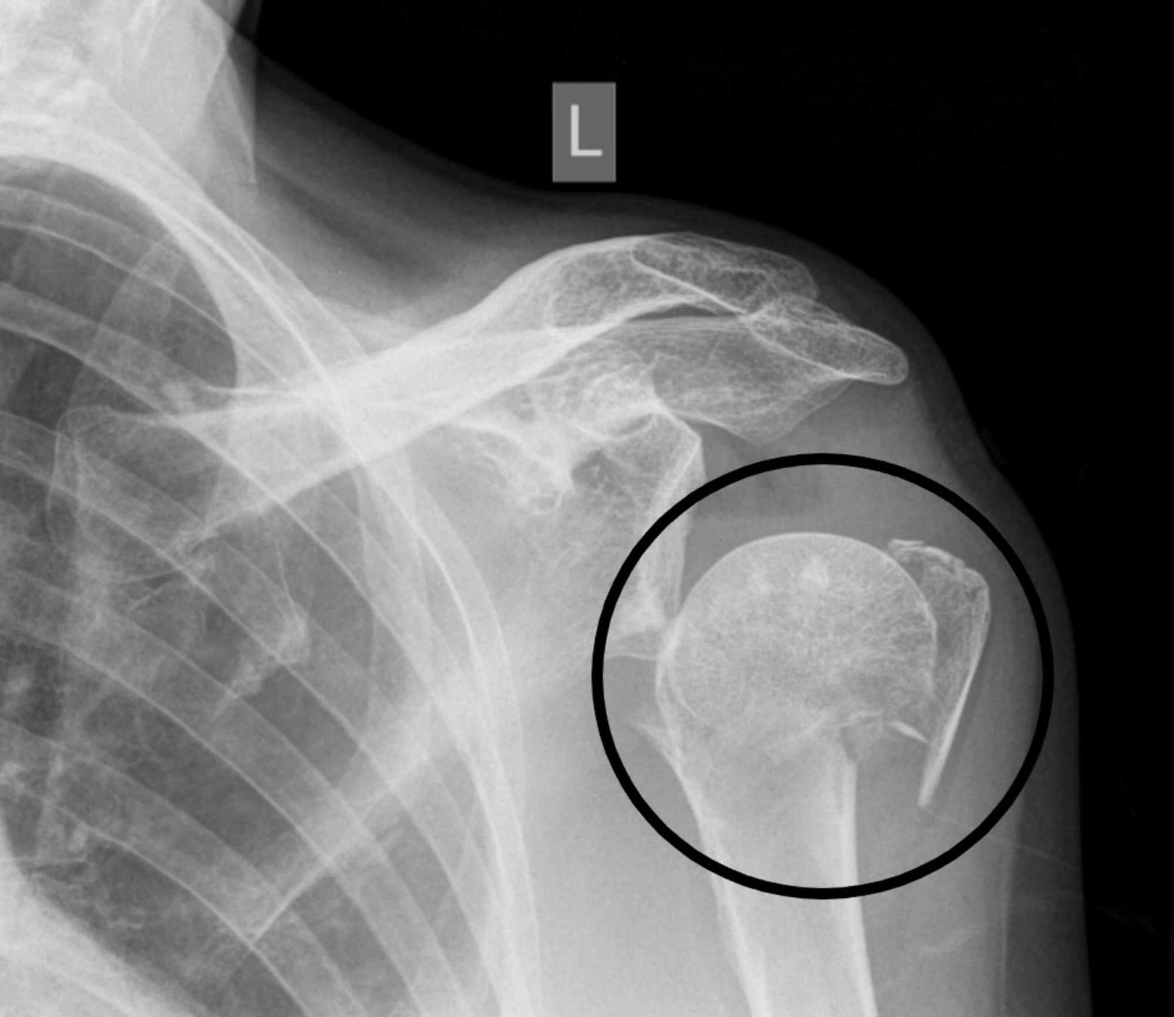
Three-part proximal humerus fracture (encircled)

**Figure 2:**
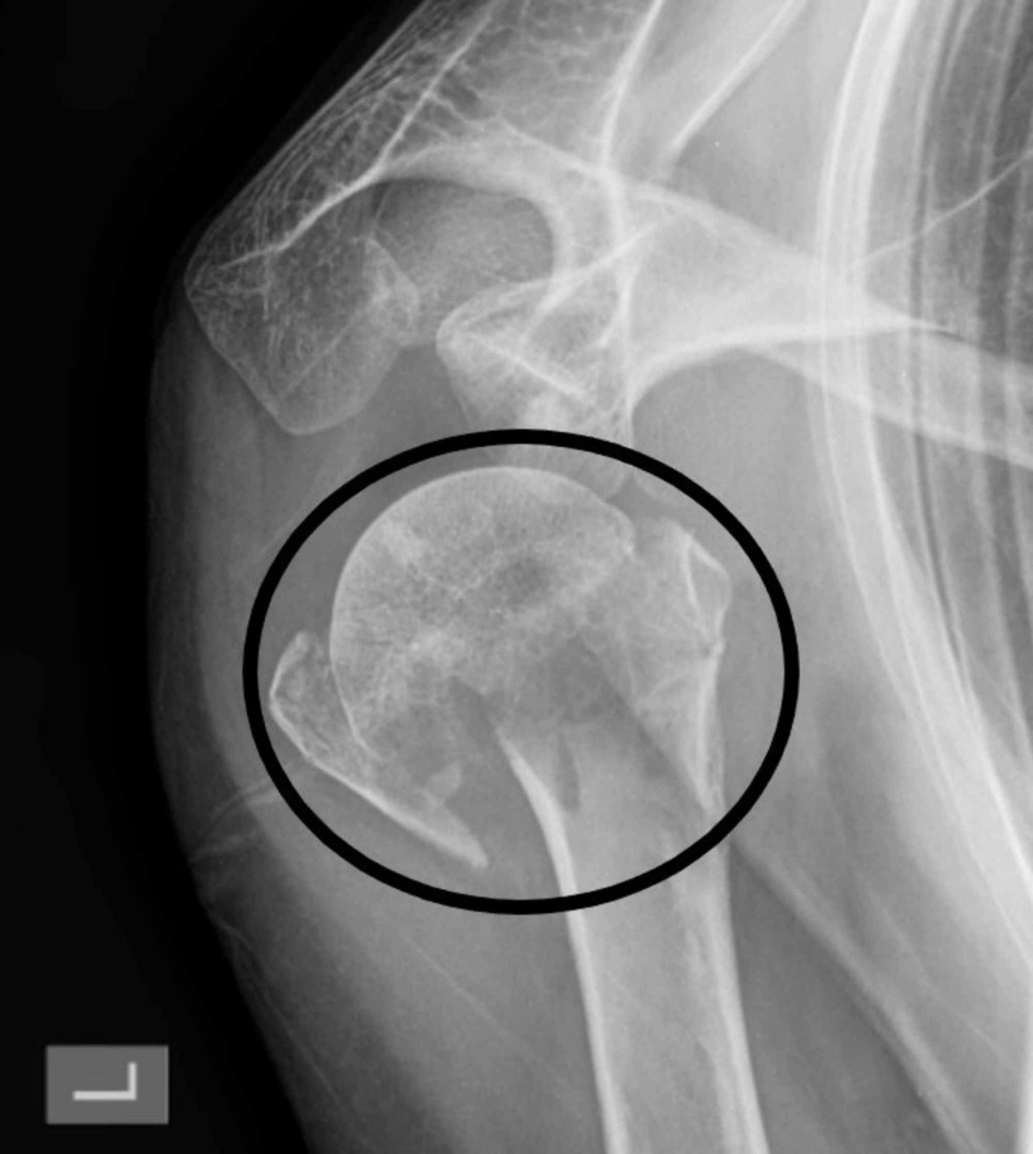
Proximal humerus fracture before reduction and fixation (encircled)

For the percutaneous fixation, the usual indications are (Figures 1, 2):

- Surgical neck +- GT fracture

- Two- or three-part fracture

- Non-head splitting fracture

- Neutral or valgus fractures

There are two fixation devices used for percutaneous fixation in our technique:

- K-wires (2 mm)

- Suture anchor device

In usual percutaneous fixation, only k-wires are used but in our technique, we employ both devices.

So when we are satisfied with the position of fracture and its reduction, then we mark a line over the deltoid region about 5-7 cm from the acromial edge as a landmark of the axillary nerve.

**Figure 3:**
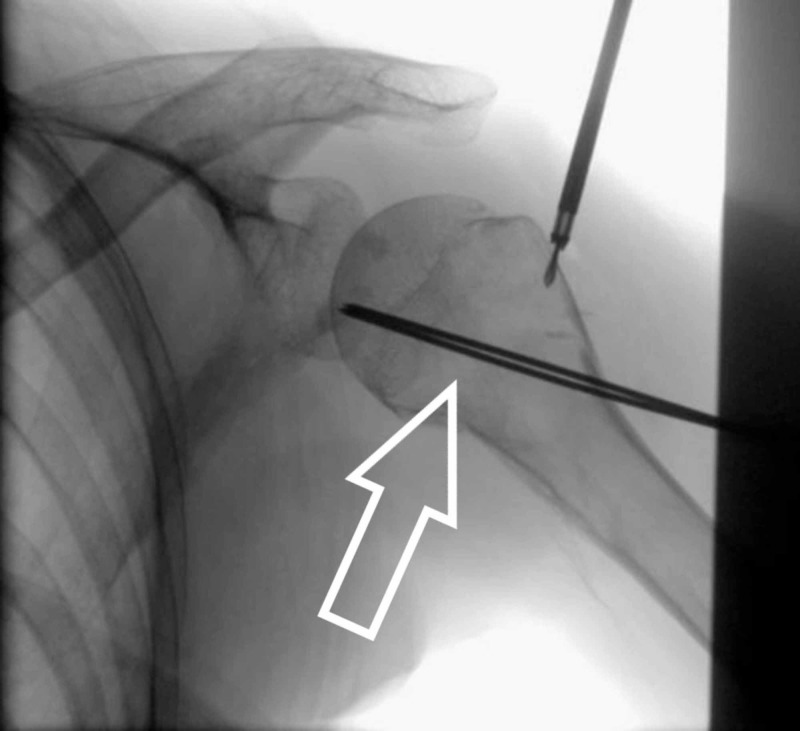
Arrowhead showing K-wire fixation

Then small stab incisions are made and the track is made over lateral upper arm for k-wire traversing with the help of artery clip. It is emphasized that we get to avoid the path of the axillary nerve. Under image guidance we use two 2-mm threaded k-wires for fixation. We use sleeves for k-wires and after making sure our trajectory we drill in the k-wires (Figure 3). Once we are happy with the position of k-wire then we move to employ the second device.

**Figure 4:**
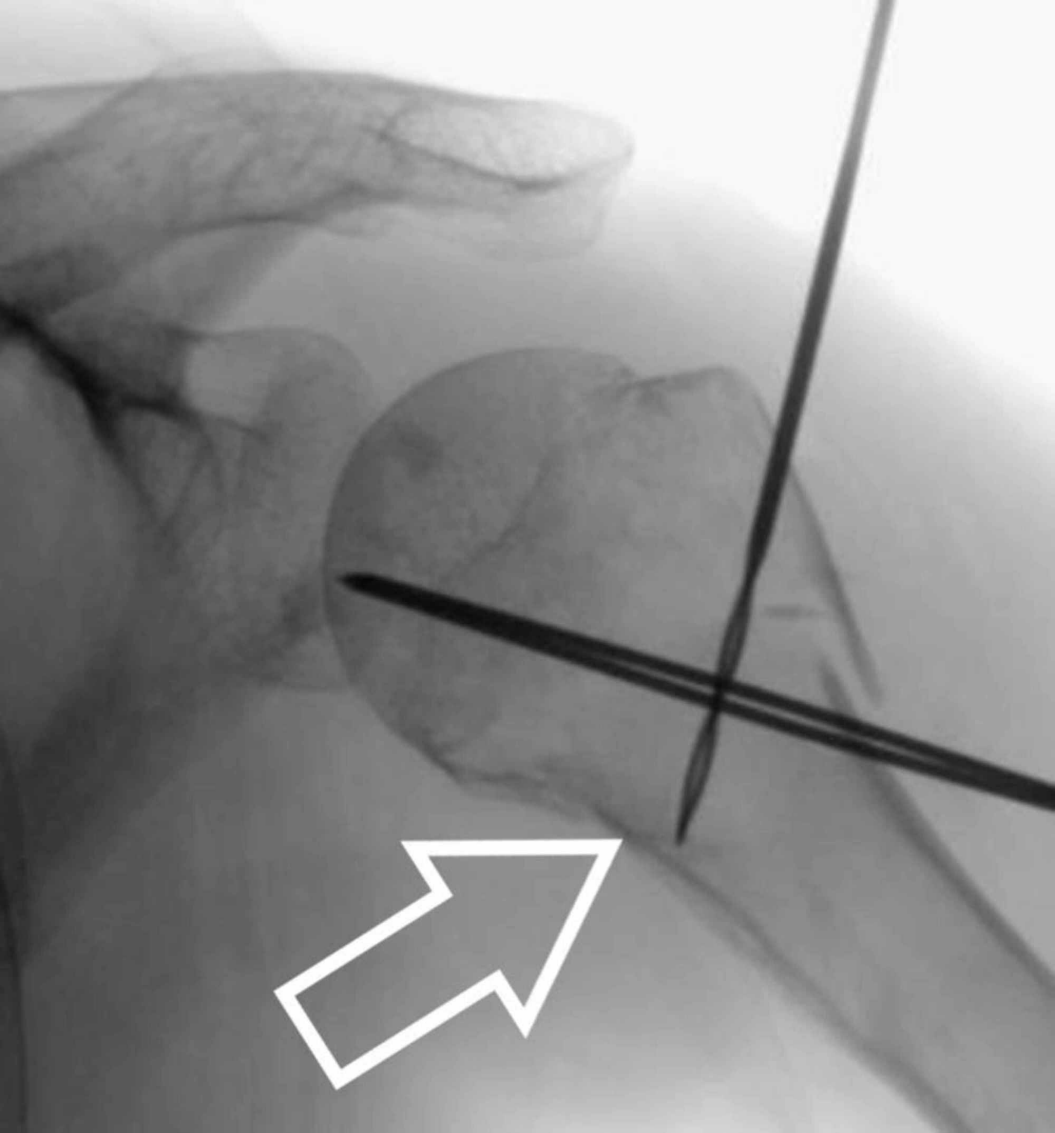
Arrowhead showing drilling for anchor device

We make a small stab incision over the greater trochanteric region of the humerus and use the artery clip to make a path for the insertion of our suture anchor device. Under image guidance, we drill for the anchor suture device aiming for the calcar region (Figure 4).

**Figure 5:**
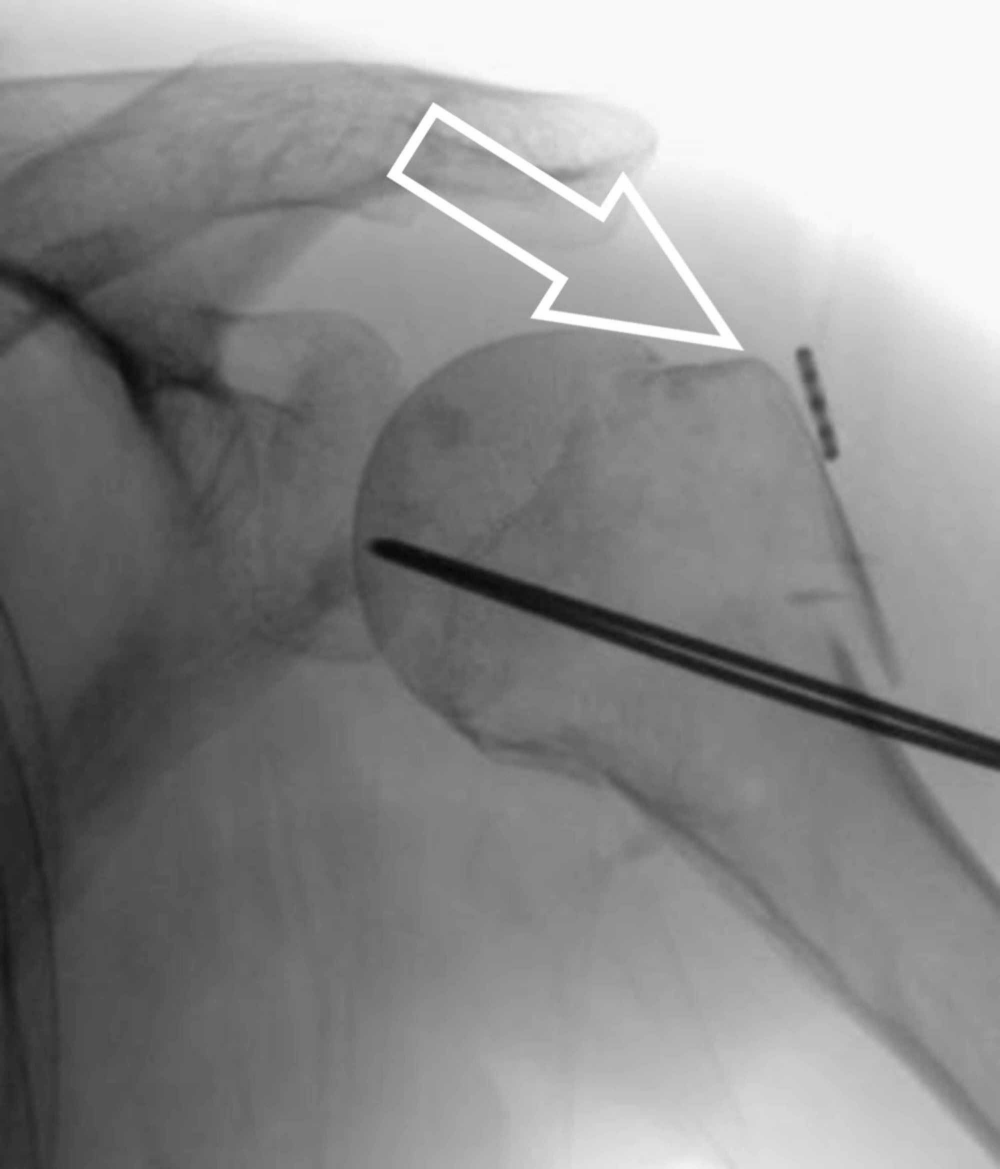
K-wire and anchor fixation (Arrowhead showing anchor device button)

**Figure 6:**
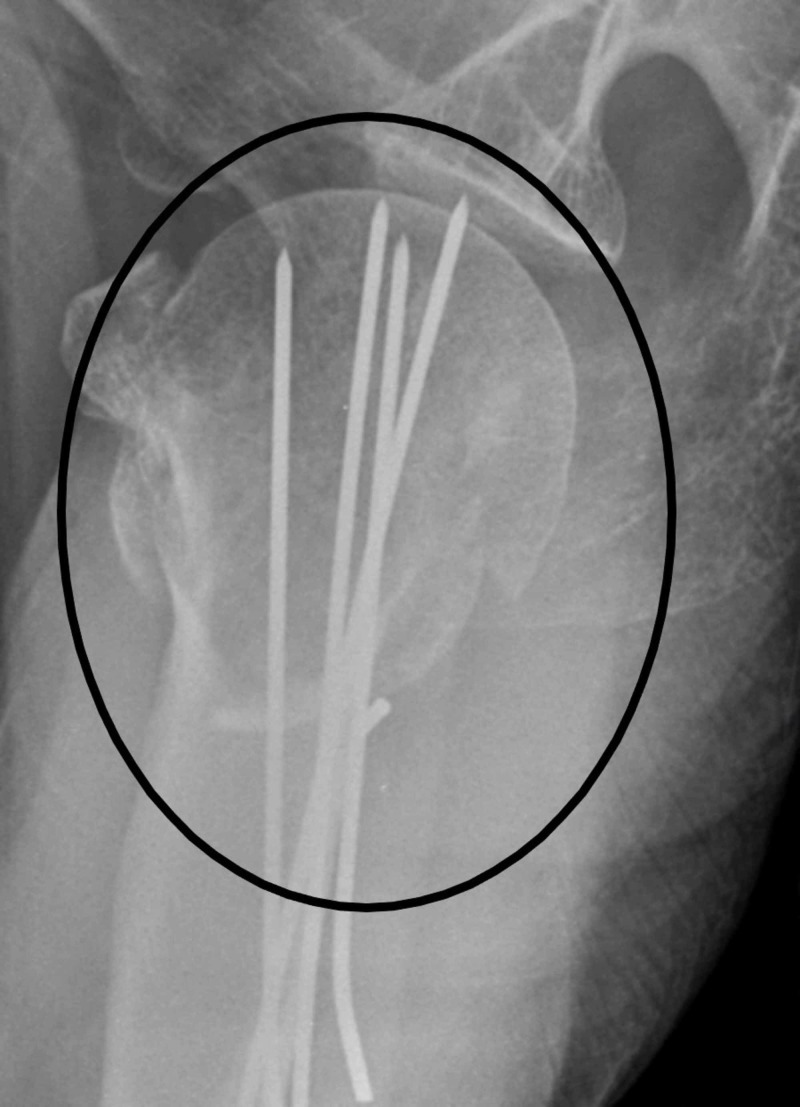
Lateral X-ray view of fixation (encircled)

Once we have drilled up to satisfaction, we introduce the suture anchor device. The device has all-suture anchors that show impressive strength whilst reducing the iatrogenic damage caused by insertion. Once the tip of the suture anchor has gone passed calcar, the ends of sutures are started to be tightened and the tip of the device expands rapidly and blocks against the hole on the other side of calcar (Figure 5). The sutures are tied and buried under the tissue as close to GT as possible (Figures 5, 6).

**Figure 7:**
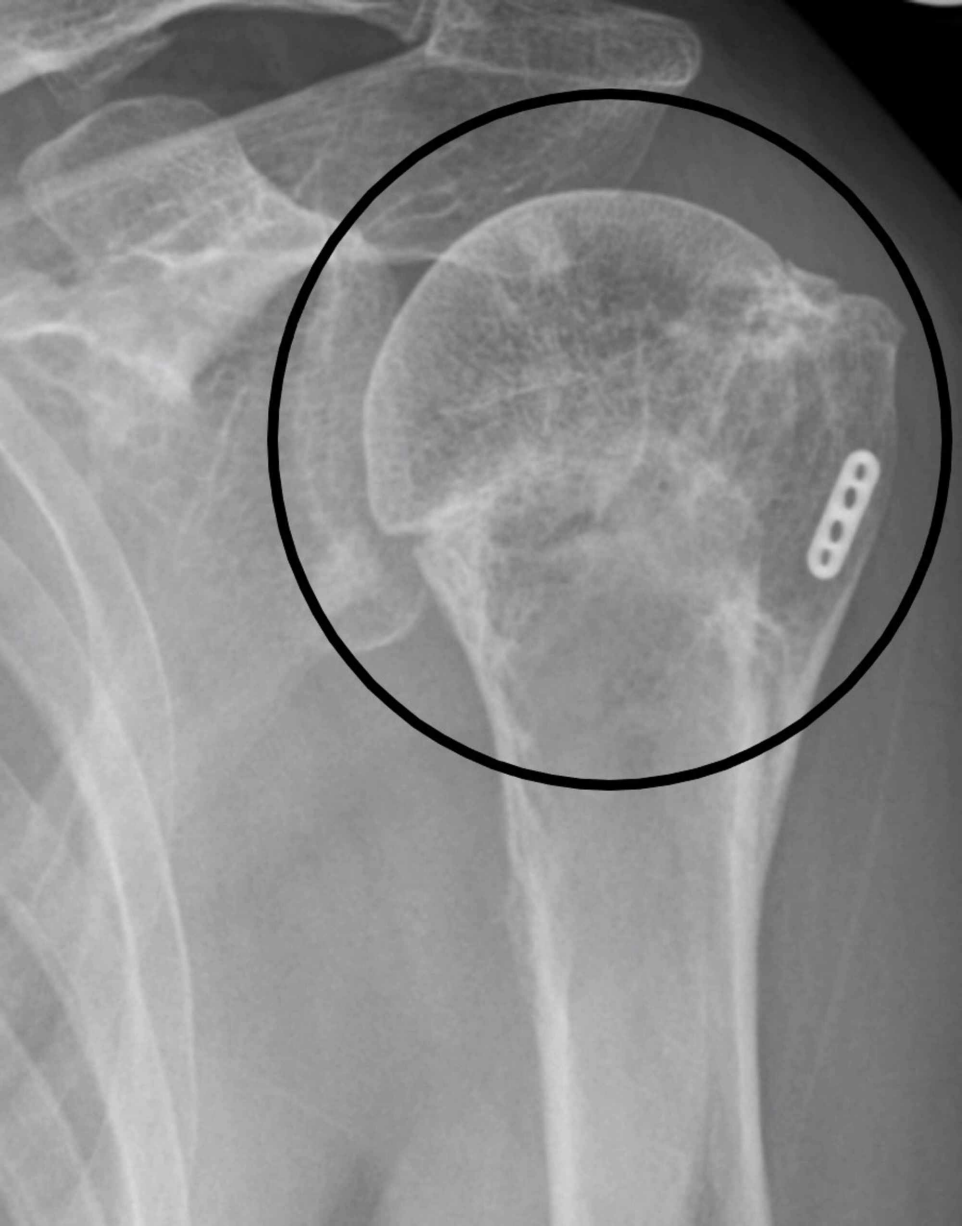
X-ray at three months (encircled healed fracture)

Once happy with the position of suture anchor device the k-wire ends are cut as well and the tips are buried under the skin. The incisions are so small that steristerips are enough for them. The patient is given a sling for comfort. The K-wires are removed in the outpatient department at six weeks. The patients are followed up at six weeks, three months and finally at six months and then discharged if the patient and surgeon satisfied (Figure 7).

## Discussion

The benefit of this technique is that firstly it is less invasive. The integrity of skin and tissues is respected [2].

Secondly, this is cost effective as compared to plate fixation. Thirdly, less chances of injuring adjacent neurovascular structures [3]. Lastly, the common problem of screw cut out is not an issue in this technique.

Although this fixation method has many advantages, still there are some complications as well like any surgical procedure [4,5]. Most common complications which can happen are k-wire related. Firstly, k-wire migration can happen. Secondly, the pin site infection is a known complication.

## Conclusions

The surgical techniques are evolving with the passage of time. It is preferred to adopt the minimalist approach in surgery as it has multiple benefits. The pre-conditions of any successful innovation in surgical procedures are that they should be less invasive, more successful, cost-effective, quick rehabilitation and help in the early resumption of maximum functional capacity. Like any other technique, our technique has advantages and disadvantages as well, but we just wanted to introduce our technique as something which offers lesser invasive, lesser complication, cost-effective and successful technique.
